# The History of *In Vivo* Tuberculin Testing in Bovines: Tuberculosis, a “One Health” Issue

**DOI:** 10.3389/fvets.2018.00059

**Published:** 2018-04-09

**Authors:** Margaret Good, Douwe Bakker, Anthony Duignan, Daniel M. Collins

**Affiliations:** ^1^Independent Researcher and Private Consultant, Dun Laoghaire, Co. Dublin (previously affiliated with the Department of Agriculture, Food and the Marine, Dublin), Ireland; ^2^Department of Animal Health, Faculty of Veterinary Medicine, Complutense University of Madrid, Madrid, Spain; ^3^Department of Agriculture, Food and the Marine, Dublin, Ireland; ^4^Centre for Veterinary Epidemiology and Risk Analysis, UCD School of Veterinary Medicine, University College Dublin, Dublin, Ireland

**Keywords:** tuberculosis origins, tuberculosis in a social context, zoonotic tuberculosis, tuberculin test in cattle, One Health and tuberculosis, tuberculosis, bovine TB

## Abstract

Tuberculosis (TB) is more than 3 million years old thriving in multiple species. Ancestral *Mycobacterium tuberculosis* gave rise to multiple strains including *Mycobacterium bovis* now distributed worldwide with zoonotic transmission happening in both directions between animals and humans. *M. bovis* in milk caused problems with a significant number of deaths in children under 5 years of age due largely to extrapulmonary TB. This risk was effectively mitigated with widespread milk pasteurization during the twentieth century, and fewer young children were lost to TB. Koch developed tuberculin in 1890 and recognizing the possibility of using tuberculin to detect infected animals the first tests were quickly developed. Bovine TB (bTB) control/eradication programmes followed in the late nineteenth century/early twentieth century. Many scientists collaborated and contributed to the development of tuberculin tests, to refining and optimizing the production and standardization of tuberculin and to determining test sensitivity and specificity using various methodologies and injection sites. The WHO, OIE, and EU have set legal standards for tuberculin production, potency assay performance, and intradermal tests for bovines. Now, those using tuberculin tests for bTB control/eradication programmes rarely, see TB as a disease. Notwithstanding the launch of the first-ever roadmap to combat zoonotic TB, many wonder if bTB is actually a problem? Is there a better way of dealing with bTB? Might alternative skin test sites make the test “better” and easier to perform? Are all tuberculins used for testing equally good? Why have alternative “better” tests not been developed? This review was prompted by these types of questions. This article attempts to succinctly summarize the data in the literature from the late nineteenth century to date to show why TB, and zoonotic TB specifically, was and still is important as a “One Health” concern, and that the necessity to reduce the burden of zoonotic TB, to save lives and secure livelihoods is far too important to await the possible future development of novel diagnostic assays for livestock before renewing efforts to eliminate it. Consequently, it is highly probable that the tuberculin skin test will remain the screening test of choice for farmed livestock for the considerable future.

## Origins of Tuberculosis (TB)

Tuberculosis is an ancient disease, found in relics from ancient Egypt, India, and China. Gutierrez and colleagues thought it likely that an early ancestor of *Mycobacterium tuberculosis* was present some 3 million years ago in East Africa, and they propose that it may have infected the great apes and ancestral man at that time. TB is largely preventable, treatable, and even curable since the 1950s yet, thousands of years after it ravaged ancient cultures, three people die of TB every minute in the twenty-first century (1.5 million in 2014), and TB continues to thrive despite the fact that the mycobacteria that cause it can only multiply and propagate inside a host (1–4).

Genetic analyses of TB mycobacteria and molecular clock evolutionary analysis dates the ancestral TB strain to approximately 40,000 years ago, which coincides with the period when anatomically modern humans were traveling outwards from Africa to settle in Europe and Asia. This ancient strain split into two major lineages between 10,000 and 20,000 years later and spread worldwide with the expansion of human populations (1, 4, 5). In ancient Greece, Hippocrates saw the disease in his patients, evidence of TB was found in scarred skeletons at various places around Europe and Asia and, during the 1990s, *M. tuberculosis* DNA was extracted from lesions on 1,400-year-old human bones found in Europe and Borneo (5). Soon after that, TB was identified in tissues from a Peruvian 1,000-year-old mummy, thus it became apparent that the disease had arrived in the Americas long before the European colonists (5, 6). The strain found in the Peruvian mummy, differed from the most prevalent strains in modern South America, being most closely related to a strain found in seals, leading scientists to theorize that seals initially picked up *M. tuberculosis* when breeding on African beaches before transporting and transmitting it from Africa to South America (7). Genetic studies of modern animal and human TB strains from around the world suggest that some sequences were shed, approximately 6,000 years ago, from a progenitor strain of one lineage of the human-adapted *M. tuberculosis*, that ultimately gave rise to what we now term the *Mycobacterium tuberculosis* complex (MTBC), and from which *Mycobacterium bovis* evolved. This, in evolutionary terms, may be associated with early farming and animal domestication between 10,000 and 15,000 years ago (5, 8). However, there are also suggestions that ancient cattle TB, with a Holarctic pattern, had reached North America some 20,000 years ago, before bovine domestication, indicating that we may not understand the entire TB spread scenario (9). Rothschilde et al. reported finding lesions resembling TB in the bones of a Pleistocene long-horned bison (*Bison cf. antiquus*) radiocarbon dated as being “17,870 ± 230 years old”. Spoligotyping of these lesions revealed pattern plots that contained MTBC segments that cannot be assigned to any individual modern species of the MTBC based on existing spoligotyping data, but which are closest to the *M. tuberculosis* group and not associated with modern *M. bovis* or *Mycobacterium microti* (9).

## TB in a Social Context

Outbreaks of TB in humans peaked with a prevalence as high as 900 deaths per 100,000 of population between the eighteenth and nineteenth centuries as primarily farming, rural societies in Europe and America became industrial and urban when, during the industrial revolution, field workers moved to the cities in search of work. This rise in TB deaths reflected the impact of poverty, malnourishment, primitive sanitation, poorly ventilated housing, and overcrowding (10, 11).

Driving forces, like those at work in humans, influenced the prevalence of TB in cattle in the eighteenth and nineteenth centuries. In tandem with the migrating rural human population, in an era without refrigeration, milking cows also moved to cities. There they were kept in close confinement with poor ventilation which led to increased TB prevalence in cows and consequently *M. bovis* in milk. TB prevalence in cattle was further aggravated by the subsequent progressive amplification of cattle production (10). The zoonotic risks of *M. bovis* in milk from an infected bovine population were already known in 1895 when it was reported that milk from animals with TB contained tubercle bacilli visible microscopically even though the cows were lacking detectable lesions of the udder and that such milk could transmit disease orally to guinea pigs, rabbits, pigs, and calves (12). In New York, milk pasteurization began in 1912 and, in the decade following when 50% of milk was consumed pasteurized, the non-pulmonary TB death rates declined by 50%. Furthermore, whereas previously 50% of tuberculous neck glands had been confirmed as *M. bovis*, once pasteurization became the norm only 6 of 50 such glands confirmed as *M. bovis*, 5 of which were found in people who drank raw milk (13). During the late nineteenth and early twentieth centuries, it became increasingly clear that TB could be transmitted through food, particularly milk. By 1914, TB specialists agreed that most human cases of TB in the stomach, neck glands, and throat had been transmitted by the consumption of infected milk (14). *M. bovis* of bovine origin was estimated to be responsible for approximately 15,000 deaths in the US in 1917; three times as many as die from all food-borne illnesses today (15).

It is estimated that in Great Britain (GB), 6% of human deaths due to TB were due to *M. bovis* before the introduction of any effective bovine TB (bTB) control programmes (16). It is now difficult to credit that by championing a campaign for clean and honest milk without adulteration, e.g., added untreated water, or germs of infectious diseases, and the adoption the American style system of milk certification for cleanliness (introduced in the District of Columbia in 1904), Wilfred Buckley became the “*bête noire*” and the enemy of farmers and the milk trade (17). Nevertheless, despite human health implications and attempts by three Royal Commissions, between 1890 and 1911, to define the disease as a public health and food safety problem, it appears to have been the economic consequences of bTB that led to attempts to eradicate the disease in British cattle (18). Discussions on the grading of milk and also pasteurization had been ongoing in GB from around 1914. However, opposition to changes that would have reduced the risk of TB to consumers both from ideologues and vested interests, including the farming industry and parts of the milk trade ensured that the political parties in power took no decisive action and compulsory pasteurization was delayed (17) even though mandatory pasteurization of milk had been introduced in New York city as early as 1910 (19). Action to eliminate bTB was regarded as “too big a problem,” “damaging to the farming industry,” with “uncertain science” but without any concept of the necessity to act with a duty of care to the consumer or the precautionary principle (17). It was generally accepted that 40% of the British milking herd was infected with TB in the 1920/1930s, nevertheless, pasteurization was only gradually introduced by the industry between the two world wars and then only to increase the shelf life of retail milk (17). It seems strange now that at the time there was no appreciation of the need to engender the trust of consumers in the food chain or that this is compatible with the interest of farmers. It was not until 1935 that it became policy to develop “Attested herds” for TB freedom and, in 1947, Francis reported 1% of tuberculous cows as having TB of the udder which represented roughly 0.5% of all cows in GB at the time (8). It was not until the 1950s that compulsory slaughter of TB-infected cattle was enforced in the UK with the nationwide bTB eradication campaign (17).

In 1992, Hardie and Watson (20) noted that milk pasteurization was not widespread in the UK until the 1930s. It reached approximately 50% of the population by 1939 while raw milk was still supplied from non-attested herds in 1960. Hardie and Watson (20) reference W. A. Lethem (1955. Milk-borne tuberculosis, 1921 to 1953. *Monthly Bulletin of the Ministry of Health and the Public Health Laboratory Service* 14, 144–145) to say “In 1955, Lethem used deaths from abdominal tuberculosis in children under 5 years as an index for *M. bovis* infection. In this age group, this form of tuberculosis was thought to be almost entirely due to ingestion of milk contaminated with *M. bovis*. The fall from 1,107 deaths in 1921 to 12 deaths in 1953 was much more marked than the equivalent reduction in deaths from other non-pulmonary forms of tuberculosis. This was attributed to the development of ‘safe’ milk in the intervening years, due to a combination of pasteurization and control of *M. bovis* infection in cattle.” They also demonstrate that in an area where bTB is endemic, raw milk may still be a risk by reporting a clinical outbreak of *M. bovis* affecting three school children in 1959 and attributed it to contaminated milk from a herd infected following a tuberculin test (20).

The benefits of pasteurization of milk, when bTB levels are high, to reduce TB infection particularly in young children are well established. In The Netherlands, pasteurization of milk had spread in the 1940s resulting in a reduced mortality rate per 100,000 in children under 4 years from 3.03 in 1934 to 0.76 in 1945 and, likewise, in children between 5 and 14 years from 2.16 in 1934 to 0.92 in 1945 per 100,000 (21). This represented a remarkable decline when you consider that this period coincided with World War II in Europe when the incidence of TB in people increased in Europe, including in The Netherlands, and among USA military personnel, before falling again (10, 11, 22).

Zoonotic *M. bovis* TB is still a problem even in the developed world. For instance, in the USA, human infection with *M. bovis* has been mostly, but not completely, eradicated with milk pasteurization combined with, the culling of herds with skin test-positive animals from about 1917 (15). Human *M. bovis* cases usually account for <1% of all human isolates in the USA, primarily in immigrants, and predominantly located in extrapulmonary sites (cervical and mesenteric nodes, the peritoneum, and the genitourinary tract), although *circa* 50% of adults will only present with pulmonary TB (23). An analysis of TB trends from 1980 to 1991 (23) and from 1994 to 2005 (24) in San Diego County demonstrated that the annual *M. bovis* culture-positive rate as a proportion of all TB cases increased annually, from 3% in the earlier study to 5% in 1994 and to 11% in 2005 (*p* < 0.001). Between 1994 and 2005, 8% (265/3,291) of culture-positive cases were confirmed with *M. bovis* and 92% (3,026/3,291) with *M. tuberculosis* (24). However, the incidence of *M. bovis* was higher in children <15 years (45%) than in adults (6%). The study authors cautioned that this may be an underestimate as culture was only successful in 80% of cases under national and local clinical case definitions with unsuccessful culture overrepresented in those otherwise most likely to have *M. bovis* TB. Usually, only adults died during treatment with mortality in *M. bovis* cases twice as high as in *M. tuberculosis* cases. Persons of Hispanic ethnicity accounted for >96% of the *M. bovis* TB cases, with 60% occurring in those of known Mexican origin. In addition, factors associated with *M. bovis* TB included having extrapulmonary disease with a normal chest radiograph suggesting that the source of infection was most probably oral with consumption of Mexican unpasteurized dairy products identified as the major risk factor. The authors suggested that to ensure elimination of this zoonotic transmission, regulation of unpasteurized dairy product production, and eradication of bTB in dairy cattle was required in the long term (24). Similarly, in Ireland in 2005, a case was detected involving two children infected by a cow with only a high somatic cell count as evidence of the presence of *M. bovis* in her milk (25). In this case, however, all milk supplied from the herd was pasteurized, and thus raw milk consumption was only a zoonotic risk to the farm family. It was the detection, at a routine Single Intradermal Comparative Tuberculin Test (SICTT), of positive calves that had also been fed raw milk on the farm which was the sentinel in this case that prompted an investigation of the TB status of the family members on farm (25). *M. bovis* has also been detected from the milk of dairy goats both in the bulk milk tank and in milk from an individual TB-infected goat (26). The risk of zoonotic TB is equally applicable to raw milk products from any species in a country or region with endemic *M. bovis* and hence regulation of unpasteurized dairy product production, and the elimination of TB in livestock is a long-term requirement to eliminate this source of infection. Indeed, in an environment with a high bTB prevalence, with both *M. bovis* and *M. tuberculosis* being detected in milk constituting a zoonotic risk (27), and in keeping with the goals of the WHO, and as already suggested by Soxhlet in 1886 (28), should all agencies insist on the pasteurization of milk? This would help to reduce infant exposure to pathogenic mycobacteria, would improve human health, would reduce hospital admissions and treatment costs associated with TB, and would reduce avoidable deaths.

Milk is not the only source of zoonotic TB in humans. For example, in Michigan in 2008, two humans were detected with the genotypically consistent strain of *M. bovis* circulating in Michigan’s white-tailed deer. This confirmed that recreational exposure to deer is a risk for infection in humans; therefore, hunters, trappers, taxidermists, venison processors, and venison consumers would potentially be at risk (29). Indeed, interspecies transmission of *M. tuberculosis*, human–animal–human, is a public health concern, especially with close human–animal interaction, notably in places such as circuses, exotic animal facilities, and zoos where there may be contact between TB-susceptible animals and humans (30–37). Where no effective eradication programme operates in cattle, the routine isolation of *M. tuberculosis* from multiple cattle raises the possibility of human-to-cattle-to-human transmission and the specter of increased zoonotic risk if *M. tuberculosis* strains adapt for bovines or other animals. Such findings underline the importance of adopting effective TB control and eradication programmes in humans and livestock alike (38–42). Indeed, the threat from zoonotic TB resulted in the adoption of a resolution by the OIE in 1983 which called for the eradication of *M. bovis* for both public health and economic reasons (43).

Tuberculosis in humans, without any animal involvement, still exists in Europe. A 50% rise of TB prevalence in London from 1999 to 2010 led to London being described by The Telegraph as the TB capital of Europe. At the time, the incidence of TB cases being detected in London rivaled or even exceeded that of Rwanda, Eritrea, Iraq, and Guatemala (44). The most at-risk groups were prisoners, drug users, the elderly, the homeless, refugees, migrants, and those marginalized by society. It is argued that the foreign-born component did not all enter GB having been previously infected but rather that they joined at-risk communities including the poorest in terms of housing, nutrition, and economic status. This reflects that, even in the modern era, TB still maintains its relationship with deprivation (44). The advent of HIV infection led to a dramatic resurgence of TB in humans (11). Today, notwithstanding the development of advanced screening, diagnostic, and treatment methods, one-third of the world’s population, or over two billion people, are considered to be TB infected (3, 45). In 2014, 1.5 million people including 0.4 million HIV-positive, died of TB; 140,000 of these deaths were in children (3, 45). Worldwide, it was estimated that during 2014 some 9.6 million people would have fallen sick as a new case of TB (including 1 million children) and yet fewer than two-thirds (63%) of that number or only 6 million were reported to the WHO. This means that there is a 37% worldwide shortfall in diagnosis and/or reporting of new cases. Globally, there has been a gradual reduction in the number of incident cases with a TB prevalence rate which is 42% lower in 2015 than in 1990. However, there were marked differences between high and low TB incidence countries with the African Region having 28% of the world’s cases in 2014 and the most severe burden relative to population. There were 834 cases in South Africa and 852 in Lesotho per 100,000 population, i.e., an average 281 cases per 100,000 people in the region or more than double the global average of 133 (3, 45).

## TB in Cattle

Although cattle are usually regarded as the true hosts of *M. bovis*, TB due to other members of the MTBC, mainly *M. bovis* or *Mycobacterium caprae* but more recently also *M. tuberculosis*, has been reported in many other species of domesticated and wild animals and remains a significant zoonosis (30–42). As with humans, TB in animals is contagious and spreads by contact. The usual route of infection is by inhalation, but oral infection also occurs. Disease progression is protracted, taking months or years to kill an infected animal. In the interim, transmission occurs before clinical signs manifest (46, 47). Symptoms, when evident in bovines, include the following: progressive weight loss, loss of appetite, intermittent cough, swollen lymph nodes, weakness, low-grade fluctuating fever, and diarrhea (48). Infection also leads to less obvious effects such as a reduction in milk yield of 10–20%, reduced fertility, lighter (reduced value) carcase with carcase condemnations at slaughter and restrictions on markets (49). In some animals, lymph nodes, such as the retropharyngeal and others, enlarge and may rupture and drain; if superficial lymph nodes are involved then the drainage will be evident. Swollen lymph nodes may also obstruct blood vessels, airways, or the digestive tract. When involving the digestive tract, bloating, periodic diarrhea, and/or constipation may be seen. In the terminal stages, extreme emaciation and acute respiratory distress may occur. TB can be a major cause of economic loss for both individual livestock owners and countries. Observation of symptoms becomes less evident once an eradication programme, including live animal testing and removal of those infected, commences (46).

## Tuberculin Test Development

In 1720, Benjamin Marten proposed that a microscopic living being able to survive in a new body was the cause of TB, this “being” was termed an *animacula* (50). However, there was wide disbelief of this proposal. In 1882, 162 years later, Koch demonstrated that it was true when he isolated the “tubercle bacillus” (10, 11, 15, 51). At the same time Koch declared that the tubercle bacilli and the human and bovine forms of TB were identical. Koch’s declaration apparently ignored the 1868 work of Jean Antoine Villemin, a French doctor, who described the greater virulence of bTB in rabbits as compared with human TB (15). In 1890, Koch cultured the bacillus in a 5% glycerol broth, subsequently evaporated over a steam bath to one-tenth of its volume and filtered. The resulting filtrate, Koch’s Old Tuberculin (KOT), contained the soluble fraction of the tubercle bacillus in a 50% glycerol solution. Koch, who himself had TB, demonstrated the properties of KOT, developed as a means of treating and preventing TB; and, having injected himself with his tuberculin, observed that he developed “an unusually violent attack of ague and rise of body temperature”; he also observed that subcutaneous injection in many tuberculous patients had elicited systemic reactions including hyperthermia (10). Almost simultaneously, the possibility of using this property of tuberculin to test cattle for TB was quickly recognized by veterinarians in Russia, Denmark, GB, and the USA, and a tool to help eradicate bTB had been found (10, 11, 15). Nonetheless, Koch initially regarded the skin reaction elicited by KOT as “not to be noteworthy and to be insignificant,” nor did he recognize its importance as a diagnostic tool (52). For a long period, it was commonly believed that “the therapeutic value of tuberculins is intimately associated with the tuberculin reaction,” as “the physiological response of the sensitized animal organism” (52). However, without the demonstration of any measurable therapeutic success it was soon discredited as ineffective (10).

In February 1891, McFadyean had commenced experiments in cattle; using clinical TB cases as subjects he proceeded to inject various quantities of tuberculin into the chest wall having first established the animal’s pulse and temperature (53). He proceeded to monitor the condition, pulse and temperature of the animals every 2 h for 36 h in total. He observed a steady ascent in temperature during the first 14 h following injection when the observed temperature peaked and remained high for a further 4 h and that, at 48 h, the temperature had returned to normal. He also recorded an inflammatory swelling at the injection site, increased sensitivity (Se) of enlarged glands and that, during the febrile phase, the pulse remained elevated with irregular heart action but that otherwise animals appeared as if healthy. He further reported a tuberculous cow which had the “most extensive tuberculous lesions” but which had shown no temperature increase despite receiving the same tuberculin doses as the other subject cases. He observed that in almost all cases “a reaction was obtained in the tuberculous animals, while in no case was there any rise of temperature in the control non-tuberculous animals.” He also remarks that “the tuberculous animals appear to have been in rather an advanced stage of the disease, but it still remains to be proved whether any discernible reaction will follow the injection of Koch’s fluid when the lesions are of small extent” (53).

Bang introduced the tuberculin test, using KOT, as the diagnostic tool of choice in the first official bTB eradication programme in cattle in Denmark during the early 1890s (54). This was the first programme on a national scale to acknowledge the diagnostic potential of the tuberculin for which Koch had previously been trying to demonstrate therapeutic qualities (51). The so called “Bang method” consisted of repeated, six-monthly tests, to identify test-positive animals. This allowed the separation of test-positive cows from test-negative cows and the culling of all open TB cases and cattle with “TB of the udder.” Thus, limiting transmission of infection *via* milk. It was quite an achievement in the early 1900s that he was able to certify the first farms to be test negative for several years and as suitable to sell “superior milk” or milk for infants. Allowing animals to be kept while “profitable” made participation affordable and was an essential factor in gaining support among farmers and dairies (54). Following reports [Ref. (55) as an example] on the achievements using this approach, the “Bang method” was internationally accepted as the major diagnostic tool in the control of bTB. Repetitive use of tuberculin tests remains the basis of all bTB control programs to this day. By the end of 1891, cattle testing using KOT was already operating extensively; and Professor Eber, a veterinarian from Berlin, reported a test specificity (Sp) of approximately 87% having collected statistics on tested cattle (15).

Palmer and Waters provide a concise and interesting review of the origins of the bTB eradication programme in the USA and the many factors of the pathogenesis and epidemiology of bTB, known or at least hypothesized (*surprisingly accurately*) as early as 1899 (15). First tests on cattle involved injecting “tuberculin” subcutaneously in the right scapular region and necessitated the veterinarian making several preinjection temperature measurements followed by regular measurements for 24 h post-injection to monitor any increase in body temperature. However, the variability in temperature change for the subcutaneous test foreshadowed difficulties associated with this application. Between 1892 and 1915, test methods began to vary dramatically, culminating in the lament that “the value of the test depended too much on the ability, competency, and experience of the examiner” (15). Despite these limitations, the subcutaneous tuberculin test, used in a test and slaughter program in the District of Columbia, reduced disease prevalence from 18.87% in 1909 to 0.84% in 1918. Unsurprisingly, the whole idea of testing cattle to eradicate bTB was not without its opponents. Some believed it unnecessary to detect bTB in the early stages of disease instead advocating only a physical examination by veterinarians to determine the high shedders for removal (*redolent of the discussions today pertaining to another mycobacterial disease—Johne’s disease*). Others, given the chronic nature of the disease, considered the US approach of test and cull too harsh, or argued that disease control should be managed by herd owners and not by government. At the same time, there were numerous rumors and misconceptions circulating about the test. The most passionate objection being that the test was inaccurate, that (*apparently*) healthy cows tested positive and diseased cows tested negative so that the test would annihilate the cattle population and result in deficits in milk and meat (15). Palmer and Waters describe how unscrupulous cattle dealers and others, including some veterinarians, specialized in circumventing or even failing to perform tests so that infected animals could be sold as having been tested (15). Even today, more than a century later, bTB eradication programmes face remarkably similar challenges including rumors as those described by Palmer and Waters (15). These include misconceptions, careless, illegal or fraudulent activities, demands for greater efficiency (less costly, easier to perform), and more effective tests (100% accurate), complaints with respect to test Sp, more so than Se, indemnity, supervision, lack of funds and of personnel.

The early 1900s saw an explosion in attempts to develop an alternative means to test cattle, presumably reflecting the demands of veterinarians and the industry generally, for a less onerous, more accurate test, not just in the USA as reported by Palmer (15) but elsewhere. In 1908, Charles Mantoux, a French physician, found that intradermally injected tuberculin was effective to diagnose TB (56). Also in 1908, Foth reported that 50% of the infected animals which had not responded to the skin test responded to the ophthalmic test (57). In 1909, Joseph gave a full account of the test in the neck and recorded the results in cattle, based upon measurements of the skin fold at the site of the injection (58). Joseph also discussed some other methods such as the cutaneous method developed by von Pirquet, where the antigen is applied very superficially, Wolff-Eisner’s conjunctival method, Escherich’s test, and that of Roemer and Joseph where the tuberculin was injected subcutaneously, and the intradermal methods proposed both by Mendel and also Moussu and Mantoux in 1908 (58). With many of the earlier methods, Joseph comments that efficacy depends on the degree of absorption and that, with a highly variable amount of antigen in the tuberculin, interpretation was unreliable. Joseph also noted that the intensity of the reaction is not directly correlated with the amount or severity of lesions and noted that recent infections appear to be associated with particularly strong reactions. He proposed that intradermal injections be made in the lateral neck region and an interpretation for the test. In addition, Joseph elaborated on the advantages of the intradermal test over the subcutaneous test being: no need to determine body temperature, a broader time window for measuring the reaction, reaction lasts longer and is more reliable, no milk drop associated with positive reactions, less antigen required and therefore cheaper (58). In the same year (1909), Römer also published two papers in support of the intradermal test setting out positive, inconclusive and negative criteria for test interpretation (59, 60). Christiansen, in 1910, also commented on the use of the caudal fold by Mantoux, Moussu, and another French author Vallée, but observed that the use of the side of the neck was more convenient and went on to describe the physical performance of the test, the nature and type of response in infected animals using the intradermal test on the neck (61). In 1910, Christiansen and Stub reported on investigations into the use of the ophthalmic tuberculin test, involving 852 animals, being apparently highly specific and easier to perform and to repeat more frequently than the usual test. Although it was not clear what they regarded as the usual test they did state that the usual test was more sensitive (62). They reported that the ophthalmic reactions were observable weakly at 6–12 h and stronger from 12 to 24 h post inoculation. However, with their tuberculin, only 50% of lesioned animals responded to the test leading them to comment on the variation in tuberculin efficacy (Se and Sp) depending on the strain and tuberculin preparation techniques used (62). Following on from the production of KOT several other tuberculins were produced; the *Bacillus* emulsion, the broth filtrate, and the tuberculin residue, all standardized to a definite amount (in milligrams) of solids per volume (52). In 1914, Haring and Bell lamented with respect to the subcutaneous test in California, where it was still being used, that “it cannot be applied satisfactorily to young calves or to wild range cattle, while during the hot season in some of the interior valleys the test has been unsatisfactory even when applied to docile dairy cows” (63). The demise of the subcutaneous test and the adoption of the intradermal test, using the caudal fold method as the screening test for cattle in the USA was imminent. The caudal fold test was increasingly used from 1917 and was adopted as the official means to test cattle in 1921 (15). Further details of these tests and other material on the early use of tuberculin in diagnosis and treatment of TB, from the late 1800s to *circa* 1913, may be found among the forgotten books (64).

In his 1934 President’s address to the Royal Society of Medicine, Buxton commented on the various substances referred to as “tuberculin” eliciting either a systemic or local reaction depending on how they were administered (65). He went on to discuss the intradermal tuberculin reaction saying that “the intradermal reaction may be obtained on any part of the surface of the body, but certain regions are obviously to be preferred.” Buxton noted that Moussu and Mantoux had suggested the subcaudal fold in cattle which is still used extensively, especially in the USA while others (Ligniéres, Roemer, Joseph, Bang, Jensen, and Christiansen) preferred the side of the neck. Buxton elaborated on the highly specific nature of the tuberculin reaction, that it “is capable of indicating the presence of tuberculous infection in little short of 100% of infected individuals” while at the same time recognizing that not every case of TB in either man or animals would give a positive reaction and that occasionally reactions may be “observed in apparently healthy persons and cattle.” He cautioned also that subcutaneous injection of tuberculin may cause desensitization to testing in some animals; that the comparatively low order of the skin response to tuberculin in cattle necessitates the use of a highly potent tuberculin; that there may be a temporary decrease in the reaction in the immediate vicinity of an intradermal injection extending to about a 2″ inch radius of the original injection—an obvious disadvantage in using the caudal fold owing to the restricted area suitable for injection; and that the occurrence of non-specific response can be overcome by the use of a synthetic culture medium and precipitation in the production of tuberculin. At that time, Buxton was already talking about the inheritance of a predisposition to infection as well as that relationship to the post-infection development of disease (65).

In 1942, following on from the work reported in 1939 by Buxton and Glover (66) who attributed a precision of 87–97% to the tuberculin test and recommended the use of synthetic medium tuberculin (*also better purified using precipitation methods*), the instructions to perform the SICTT in GB were that the tuberculin was to be “injected in the middle third of one side of the neck on a line parallel to the spine of the scapula, avian tuberculin is injected about 4″ below the crest of the neck and mammalian about 5″ below the avian” (67). It appears that the British had repeated, for the SICTT, much of the work done by Christiansen and Stub pre-1910, for the SIT and, in 1947, Francis confirmed that the test interpretation for the SICTT and optimal time of reading was “based on a very large number of trials followed by postmortem examination” (8). An allowance of ±4 h for reading on either side of the 72 h is included in the EU Trade Directive (68) but not in OIE (69).

**Figure 1:**
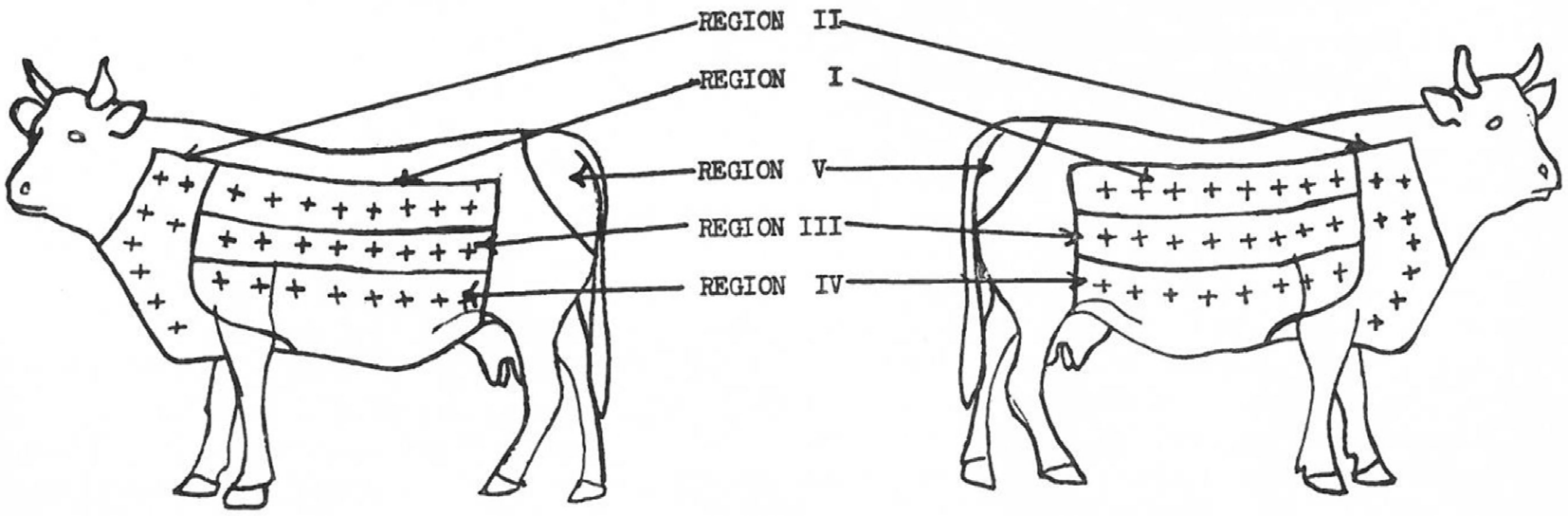
Location of skin regions on right and left sides of animals. Injection sites in each region = +. [Reprinted with permission of the AVMA (Am J Vet Res (1950) 11:301 – Larsen et al. (70))].

In 1950, Larsen et al. (70) attribute the first use of the neck as a site for intradermal injection to Joseph in 1909 and to Christiansen and Stub in 1910 who, according to Buxton and Glover (66), determined the side of the neck as optimal for the intradermal injection because this site provided the most consistent discrimination for presence or absence of TB infection. Furthermore, Larsen et al. (70) when setting out to assess if an area of skin could be found that would be more sensitive to tuberculin than the caudal fold noted that already, in 1909, Foth (57) had pointed out the deficiencies in the use of the caudal fold. They assessed site Se to johnin (a diagnostic agent for *Mycobacterium avium* subsp. *paratuberculosis* infection, analogous to tuberculin) and tuberculin in steers sensitized to either to paratuberculosis or to TB delineating five regions on each side of each animal with eight injection sites in each region except the caudal fold which had only one site (70). In total, 66 injections were made in each animal (Figure 1), and the results were analyzed. The largest reactions occurred in the neck region and showed that the skin on the neck is the most sensitive while the caudal fold was the least sensitive (70).

In 1951, Baisden et al. (71), although they acknowledged that Johnson (72), Wadley (73), and Larsen et al. (70) had individually already determined that different skin areas of cattle differ in their Se to the intradermal injection of tuberculin with the neck being the most sensitive site, reassessed the Se of the neck, the back, the upper and lower sides of cattle to intradermal injection of tuberculin. However, as well as including animals sensitized to *M. bovis* and *M. paratuberculosis*, they added animals sensitized to *M. tuberculosis, M. avium*, and *Mycobacterium phlei* to the study “because these organisms had been considered in connection with no-visible lesion cases.” The findings were that the neck was significantly more sensitive than any other site for all except the *M. paratuberculosis* sensitized group where site was not of significance. They also commented that the caudal fold was “not so likely to elicit reactions from animals of low Se” (71).

In 1959, Richie (67) also described several alternative methods of tuberculin testing, all now discarded from general use in cattle, employed in efforts to find a more efficient, effective, and less time-consuming test than the subcutaneous test which depended on multiple temperature records over time. He described a short thermal test where temperature checks were reduced, the von Pirquet test (tuberculin applied to scarified skin), ophthalmic and palpebral tests (relying on intolerance to light), increased tearing, possibly marked congestion, discharge running down the face in the tested eye, i.e., severe conjunctivitis (*still used in primates*), the Stormont test, and the vulval test. He detailed the double intradermal test (*a second dose of tuberculin injected into the swelling produced by the first usually after a period of 48 h*), also mentioned by Buxton and Glover (66) and Francis (8) as a modification of the 1910 test of Christiansen and Stub, but according to Francis commented that “it has never been satisfactorily demonstrated that it is in any way superior,” to the single test and probably tended “to reduce the Sp of the reaction.” It was not adopted anywhere as an official test until GB did so after a Medical Research Council report in 1925. The use of the word “single” in the title of the SIT and SICTT was to distinguish these tests from the earlier double intradermal test (67).

The neck was also reconfirmed as the most sensitive site by Paterson in 1959 (74) who also detailed higher Se at anterior (toward the head) sites of the neck and that Se falls off in posterior sites (nearer the shoulder) and in those adjacent to the nuchal crest; thus, he determined the middle third of the neck as the optimal injection site. This in turn was stressed by Ritchie in the same year (67), and it is why the OIE requires that the test for potency assay of tuberculin in cattle rotates through each of the 16 sites “applying eight intradermal injections per animal in both sides of the neck and employing a balanced complete Latin square design” so as to remove any site influence from the calculations as required (69, 75). In 1960, Larsen et al. reported that as the number of simultaneous injections increased, the average size of the reaction that each would elicit decreased (76). This is the reason why the selection of TB-infected animals, for use in potency assay trial performance, is confined to those that show larger reactions. It was only in 1979, on the accession of GB, Ireland, and Denmark, to the EEC that the SICTT was accepted in addition to the Single Intradermal Tuberculin test (SIT) for the Trade Directive (68).

Good et al. (77), when comparing PPDs from different manufacturers, injected control and trial avian/bovine PPDs into the same animal—the control at the anterior border of the middle one-third of the neck and the trial tuberculin at the posterior border and, by using the same tuberculin at both sites in one group of animals, demonstrated that the anterior site readings were greater than at the posterior sites and thus confirmed the earlier work of Paterson (74). Good et al. (77) also showed that it is particularly important for consistent SICTT interpretation that both injections are in the same plane parallel to spine of scapula thus confirming Ritchie’s assertion that the injections needed to be made on a line roughly parallel to the line of the shoulder (67, 77). More recently, Casal et al. looked at the effect, in cattle from officially TB free and TB-infected herds, of bovine PPD inoculation site on the skin-fold thickness increase. In TB-infected herds, there was a higher probability of positive results and larger reactions when the injections were performed nearer the head in the neck anterior area—again confirming the site effect (78). Alternative sites/methods are used for tuberculin testing in other species, e.g., pigs: the base of the ear; fowl: in the wattle; dogs and cats: monitoring body temp. However, there is very little data available on standardization of the test methodologies or interpretation criteria for species other than cattle and little or no work done on evaluation of Se, Sp, positive predictive value, safety record, or authorization criteria for use under medicines legislation and thus the reliability of tuberculin skin testing in species other than cattle is debatable.

The OIE (69) now lays down standards, applicable to cattle, for:

(1) The Delayed hypersensitivity test (i.e., the tuberculin test or the SIT) as the standard method for detection of bTB which “involves measuring skin thickness, injecting bovine tuberculin intradermally into the measured area and measuring any subsequent swelling at the site of injection 72 h later.”
(2) Performance of the SICTT where “bovine and avian tuberculin is used mainly to differentiate between animals infected with *M. bovis* and those sensitized to tuberculin due to exposure to other mycobacteria or related genera” (e.g., as used in Ireland and the UK) including providing in detail for
(3) “Test procedure—(i) A correct injection technique is important. (ii) The injection sites must be clipped and cleaned. (iii) A fold of skin within each clipped area is measured with calipers and the site marked before injection. (iv) A short needle, bevel edge outwards and graduated syringe charged with tuberculin attached, is inserted obliquely into the deeper layers of the skin. The dose of tuberculin is then injected.” (v) “A correct injection is confirmed by palpating a small pea-like swelling at each site of injection. (vi) The distance between the two injections should be approximately 12–15 cm. In young animals in which there is no room to separate the sites sufficiently on one side of the neck, one injection must be made on each side of the neck at identical sites in the center of the middle third of the neck. (vii) The skin-fold thickness of each injection site is remeasured 72 h after injection. (viii) The same person should measure the skin before the injection and when the test is read.”
(4) Tuberculin potency “The recommended dose of bovine PPD in cattle is at least 2,000 international units (IU) and in the comparative tuberculin test, the doses should be no lower than 2,000 IU each”; “In cattle with diminished allergic Se, a higher dose of bovine tuberculin is needed, and in national eradication campaigns, doses of up to 5,000 IU are recommended” and
(5) Potency assay including the necessity to assay in the target species cattle.

In performing a tuberculin test, it is important that the tuberculin used is fit for purpose (46). This was previously recognized in 1908 when it was lamented that “some of the tuberculin on the market is impotent and worthless” and Buxton also commented on tuberculin quality in 1934 (65, 79). Alas such substandard tuberculin, even unauthorized, under any recognized medicines legislation, can still be found on the market and being used to “certify” freedom from disease (80–82). Good et al. (83) compared “the impact of different potencies of a single bovine PPD tuberculin on the field performance of the” SICTT and SIT and found “a significant difference in the number of reactors detected using the high and low potency tuberculins.” In addition, “the low potency tuberculin in the SICTT failed to detect 20% of 35 animals with visible lesions,” and “11% of animals with visible lesions did not show a positive bovine response (>4 mm) and would have been negative to the SIT” with these latter animals eligible for certification as TB free for export under EU rules (68, 83). In this study, “the potency estimates from the guinea pig bioassay were imprecise” with only “limited agreement between the guinea pig and cattle bioassays” performed in naturally infected cattle (83).

Test Se and Sp should most properly only be assessed under the conditions and in the species in which the test is performed, and caution must be exercised in extrapolating Se and Sp from one environment and/or the tuberculin from one manufacturer and/or one potency, and/or one type of tuberculin test to another (46). However, the focus on “visible” and “non-visible” lesions, and on SIT/SICTT positive animals which are not confirmed as TB infected by a laboratory has resulted in doubts being cast on Sp, positive predictive value and reliability of all tuberculin tests and frequently attempts to “confirm” tuberculin test-positive animals before their removal, using tests of far lower Se. This has, in many places, had an enormous negative impact on TB control programs. In many TB-infected areas, the focus has shifted to maximizing test Sp, even in the face of active infection in a herd, to minimize the number of “non-visible” lesion animals being identified for removal instead of seeking maximum Se to prevent transmission, to rid a herd of TB as rapidly as possible, and to prevent future breakdowns. Goodchild et al. (84) provided some useful data on the Se and Sp of the SICTT in the GB bTB eradication programme namely that in GB, Sp at animal level is 99.983 (standard interpretation) and 99.871 (ultra-severe interpretation), meaning that one false positive can be expected at standard interpretation for every 4,760–7,690 uninfected animals tested; that 91.1–93.7% of reactors are actually infected with TB (i.e., test-positive predictive value); that visible lesion or culture positive results in only 30–40% of reactors “profoundly underestimates the proportion of reactors that is truly infected”; that a small majority (>50%) of NVL/culture negative Rs are infected and that at herd level the Sp is 99.2–99.5% (average test size) and 96.5% for larger (250 animals) herd tests (84). O’Reilly had already assessed the SICTT Se under Irish conditions as 91 and 98% Se (standard and severe interpretation, respectively) (85). Costello et al. obtained similar results, on repeating the study, 90.9% Se (89.6 and 91.2—standard and severe interpretation, respectively) (86). Both these studies slaughtered and examined all animals (221 and 353, respectively) involved (85, 86). Experiments to establish test Se and Sp for a particular environment are acknowledged as expensive and labor intensive and that few involve slaughter of all, including the non-reacting, cattle noting that recently infected animals are unlikely to have visible lesions or to confirm on culture (87). In Ireland, field experience evidences that only a fraction of 1% of the positive reactors to the SICTT on a national basis are false positive (88), the SICTT Sp has been calculated as 99.8–99.9% and demonstrated mathematically in an accepted non-disease-free population, as at least 99.95% (85, 89).

Research continues into the development of new, more accurate, more sensitive tests, less reliant on the measurement of the immune response *in vitro* (bovine interferon-γ release assay or antibody ELISA) or *in vivo* (skin tests) which are less subject to the vagaries of individual operative performance and subjective interpretation (90). The strategic application of the IFN-γ assay (IGRA), which in common with the tuberculin skin test reads the cell-mediated response to antigens and as such is an early-stage means to detect infection, has been found to be a useful adjunct to the tuberculin test (91–97). Thus, in more recent years, the assay has been used in European countries, in accordance with legislation, and also elsewhere to facilitate the early removal of infected animals that are otherwise negative to the tuberculin skin test in problem herds and speed up the clearance of bTB in outbreaks (68). However, the Sp of this assay has, to date, precluded its use as a screening test and research continues into the use of specific antigens to address Sp issues but, to some extent at least, this is likely to be at the expense of test Se (94, 97). While *in vitro* tests, tuberculin based or otherwise, are suitable for domestic species the logistical difficulties with their application, will vary from region to region and between different breeds and farming systems, for example, the availability of suitable laboratories with associated costs may be an issue, and the length of time taken from blood sampling to submission of samples can be critical for IFN-γ assay with laboratories likely to be some distance from infected herds. Therefore, for the considerable future, it is highly probable that the tuberculin skin test will remain the screening test of choice for farmed livestock.

Tuberculin skin tests are rarely, however, suitable for use in wild species due to the necessity to have access to the animal to read the test some days post tuberculin injection. Thus, test methods such as *in vitro* immunological tests need to be developed for use in wild or feral maintenance species as they have for bovines (87, 98). Scientific advances allow new matrices, such as high-throughput sequencing of the peripheral blood mononuclear cells to be evaluated, also the diagnostic potential of immunostaining with anti-MPT64 in various tissue specimens for *M. tuberculosis* infection (99). It remains to be seen if such tests will be added to the armory of tests in the battle against TB in any or all species in which it occurs. There are several reviews of all currently available tests, including the use of ELISA tests to detect a humoral response, therefore a later stage detection test than the IFN-γ, and the effect of a prior SIT or SICTT on the ELISA or IFN-γ response (100–107).

## Impediments to the Eradication of TB in Bovines

While bTB has been successfully eradicated in many countries, others, despite making major efforts, have been less successful (108, 109). Palmer (110) has described TB as a reemerging disease at the interface of domestic animals and wildlife, he cautioned that it will not be possible to eradicate *M. bovis*, or presumably *M. caprae*, from livestock until transmission between wildlife and domestic animals is halted, and he advises that to achieve this will require a “collaborative effort between stakeholders” (110). In 1958, Francis (111) speaking of the difficulties in final eradication of TB, also recommended that TB had to be dealt with in all species to achieve complete success. Therefore, the need to tackle TB transmission between wildlife and domestic animals is not a new concept or suggestion. It is widely accepted that for effective control of TB the disease must be addressed in all species, in which infection establishes and becomes self-sustaining (infected maintenance species) and that tackling the disease in one species alone in an ecosystem with multiple infected maintenance species will not promote a successful outcome (110). Consequently, other species sharing the environment with cattle must be risk assessed to identify potential maintenance hosts, and, where other species will constitute an impediment to final eradication of bTB, appropriate control strategies should be developed and/or adapted (112–115). The experiences in other countries with similar problems should be taken into consideration (113, 116). TB, caused by several members of the MTBC, has been reported in wildlife in several countries in Europe which are laboring to eradicate bTB (109, 117, 118). It is becoming increasingly obvious that endemic TB in wildlife populations is posing a significant constraint to final eradication of disease in cattle. Further, *M. caprae* is now recognized as not being restricted to Spanish goats, as strains of this organism have been isolated from cattle, wild boar, pigs, and humans. Its occurrence has also been reported in France, Austria, Germany and elsewhere (119).

Unsurprisingly, TB has not spread uniformly into wildlife, and it has become more of a problem in those countries and regions where bTB eradication commenced relatively late, where farming involved pasturing of cattle, where they shared the environment with susceptible wildlife, where wildlife density and behavior patterns (not necessarily the same even for the same species in all ecosystems) brought them into contact with infected cattle, and in particular where cattle were not housed. Feed supplied to cattle would have also been available to local wildlife, and this would have put wildlife and cattle into closer contact than they might otherwise have been the case (120). In other ecosystems, drought encourages the congregation of cattle and wildlife species at water holes and/or where pasture/feed is still available. Tuberculous carcases and/or entrails would, even today, be a potential source of infection for wildlife. This is particularly a problem for carnivorous and scavenging species such as lions and lynx in Africa and Spain respectively (121, 122). Thus, TB also has significant conservation implications for some species, e.g., the Iberian Lynx (*Lynx pardinus*) in Spain (123), other species in conservation areas in South Africa (121) and for lechwe antelope in the Kafue basin Zambia (124).

As disease prevalence in cattle decreases, eradication efforts are sometimes impeded by transmission of *M. bovis* and/or *M. caprae* from wildlife to cattle. In epidemiological terms, disease can persist in some wildlife species, creating disease reservoirs, if the basic reproduction rate of the disease and critical host-community size thresholds are achieved. bTB eradication efforts require elimination of *M. bovis* transmission between wildlife reservoirs and cattle where present (125). Some wildlife species, principally the badger in the UK and Ireland, the Australian possum (*Trichosurus vulpecula*) in New Zealand (but not in Australia), and water buffalo (*Bubalus bubalis*) in Australia previously, have been recognized as significant reservoirs of *M. bovis* with endemic self-maintaining infection in these species constituting a major obstacle to disease control programmes (114, 126, 127). In Australia, elimination of wild water buffalo, not a native species, and feral cattle from areas where infection was endemic was a major component of the eradication campaign and Australia is now bTB free (114, 116, 126–128). New Zealand has employed similarly strict population control measures against infected possum populations, resulting in considerable progress (117, 126, 128, 129). Michigan State, USA, had been TB-accredited-free state from 1979, with no tuberculous cattle detected for 5 years, when a hunter found a TB-infected deer in 1994. The local deer population was endemically infected with *M. bovis* and spill back was also detected in local cattle farms (130). Consequently, on-farm risk mitigation measures against the TB transmission from deer to cattle have been recommended (131). Portugal has reported wild boar (*Sus scrofa*) and deer (mainly red deer—*Cervus elaphus*), both key game wild ungulate species, as being infected with *M. bovis* or *M. caprae* in the important higher density hunting regions where TB prevalence in cattle is also highest (118). Observations in Spain showed that strains of MTBC that originated in bovines and caprines also circulate in the sympatric wildlife populations and that in addition 6 out of 11 spoligotypes resembled types described in human TB cases. The isolation of MTBC strains (belonging either to *M. bovis* or to *M. caprae*), in fenced estates, from cervids and wild boars that have not had contact with domestic livestock for at least two decades, strongly suggest that these mycobacteria are able to survive independently in these populations. Therefore, where they are TB infected, wildlife, including cervids and wild boar, need to be considered in the epidemiology and control of TB (117).

Making use of molecular detection technologies, Santos et al. demonstrated widespread MTBC contamination in environmental samples from the Iberian Peninsula. This supports the occurrence of indirect transmission as a contributor mechanism to maintaining TB in a multi-host–pathogen system (132). MTBC DNA positive samples were proportionately higher in the bTB-infected area than in presumed negative area (0.32 and 0.18, respectively) (132). In 2010, the first detection of *M. bovis* in a feral wild boar was reported in the UK in an area where the same spoligotype had previously been isolated from fallow deer, fox, wood mouse, and polecat (133). Studies under natural weather conditions in Michigan, where *M. bovis* TB had been detected in free-ranging white-tailed deer demonstrated that *M. bovis* bacteria survive sufficiently long to pose an exposure risk for cattle and/or wildlife. This strengthens evidence suggesting that biosecurity on cattle farms and efforts to eliminate supplemental feeding of white-tailed deer will decrease the risk of TB transmission among and between these populations (134). In 2016, French researchers reported finding environmental samples positive for the presence of MTBC and *M. bovis* strains in the environment of farms affected by bTB in a restricted area within the Côte d’Or region where shared genotypes of *M. bovis* circulate in a multi-host system including badgers, wild boar, and deer (135). The persistence of detection over an 8-month period, despite absence of the supposed source of infection, suggested that the DNA detected could belong to viable cells. The detection of MTBC positive signals in 10% of water samples from naturally occurring water springs and accompanying flowing water in pastures where both cattle and wildlife had access is supportive of the role of water in the dissemination of MTBC in the environment and in animal contamination perhaps even by the formation and inhalation of bioaerosols. The average prevalence of detection in badger sett soil and badger latrines was 7.3 and 7%, respectively. These were the highest prevalences detected among 356 environmental samples assessed (135). Similar work in the UK had earlier assessed that correlations between badger social group TB prevalence as determined by the qPCR assay of fecal samples from badger latrines and individual or combined diagnostic test results from trapped badgers suggested that spring was the optimum latrine sampling period, with autumn an acceptable confirmational back up with 100 and 80%, Se respectively (136). Researchers at Warwick University performed parallel qPCR of feces and culture on samples taken from badgers in areas with high bTB prevalence levels in the Republic of Ireland, which indicates that fecal shedding is a good proxy for respiratory shedding (137). In addition, the endangered Kafue lechwe antelopes (*Kobus leche Kafuensis*), in the Kafue basin in Zambia where cattle and antelope graze together during drier months, have been described as feral reservoirs of bTB (124). Wildlife management aimed at reducing the density of susceptible animals within an infected area may contribute to the control of infectious diseases in animals and, if zoonotic, their spillover to humans (125). The problems encountered in tackling disease in the various species involved in disease maintenance and interspecies transmission will be particular to the ecosystem in which they reside, and it is likely that each ecosystem will present its own challenges and indeed socioeconomic influences (46). Wildlife vaccination is an option that is being explored and the UK and Ireland are cooperating in the development of a badger vaccination strategy (138–140) in preference to continued culling, with a view to decreasing TB incidence in badgers to reduce transmission to cattle. In Spain work is ongoing into the development for a vaccine in wild boar (141). If a vaccine can be developed for badgers or wild boar then, in time, it may also be modified for use in other wild species (120).

The above examples illustrate that attributing apparent shortcomings in bTB eradication programmes to failure of the tuberculin test, as often happens, in regions or counties which harbor infected maintenance hosts may be misplaced. Rather, the true value of the tuberculin test is as an indicator of the presence of TB in the population under test. Identification of infected wildlife as the source of such infection is not the function of the test and is rather the function of a sound epidemiological investigation into the source of the TB.

## One Health

In the early years of the twenty-first century, bTB has largely been reduced to a disease of limited economic importance in the developed world, with controls causing more irritation than the disease itself. Poorer countries are facing a multifaceted impact from TB, which is not merely of significant economic impact, but which also potentially affects the health of livestock, humans, and ecosystems simultaneously and which is likely to increase in the presence of debilitating diseases such as HIV/AIDS and other factors which negatively affect human livelihoods (142). The interplay between humans, livestock, wildlife, and ecology in the epidemiology of zoonotic TB makes TB an ideal target for a One Health approach. Such an approach would enable the development of disease control programs involving both animal and human populations, and allow for expanding scientific knowledge, improving medical education and clinical care, and the development of effective disease control programs for both human and animal populations (143). One Health deals with the very essence of TB as a zoonoses—it is surely axiomatic that the transmission of disease shared between human and animal species must be addressed at multiple levels rather than focusing on humans only or specific animal species only or particular mycobacteria that can cause TB and that environmental, ecological, and sociological factors must be considered in the development of effective disease control programmes.

The One Health concept recognizes the important links between human, animal, and environmental health and provides an important strategy in epidemic mitigation and prevention. It was described by the veterinarian Schwabe (1927–2006) in his book “Veterinary medicine and human health” where he proposed a unified human and veterinary approach against zoonotic disease. The concept is, however, not new. Rudolf Virchow (1821–1902), who coined the term “zoonosis,” said “between animal and human medicine there are no dividing lines—nor should there be.” James Law (1838–1921) professor of Veterinary Medicine in Cornell educated in the Edinburgh University Medical School and Veterinary College as well as veterinary schools in France believed in “one medicine” where physicians and veterinarians should have close relations. Law’s work on TB in the USA had a profound effect on both animal and human health. Likewise, in the early 1890s, one of Bernhard Bang’s goals listed as part of the “Bang method” of TB control/eradication in bovines was to limit transmission of TB infection *via* milk, and so to specifically sell safer milk for infants (54). BCG vaccine, developed by attenuating *M. bovis*, of cattle origin, used since 1921 to protect humans from TB was developed by the French physician Albert Calmette and veterinarian Jean-Marie Camille Guérin. Their collaboration demonstrated the “one medicine” or “One Health” concept in action even though Guérin’s veterinary background and family TB problems is largely ignored (144). Basil Buxton, Veterinarian, addressed the Royal Society of Medicine in 1934 on the role of tuberculin in the control of TB in the section on Comparative Medicine (65). However, while the concept seems to have been embraced by medical and veterinary communities in the nineteenth century, it seems to have fallen into disfavor during the twentieth century when collaborative efforts between the professions diminished (144). Nevertheless, the “one medicine” concept survived and extended to “One Health” when the Washington Post, in 2003, credited William Karesh who was a veterinarian and president of the World Animal Health Organization (OIE) Working Group on Wildlife Diseases, as saying, “Human or livestock or wildlife health cannot be discussed in isolation anymore. There is just One Health and the solutions require everyone working together on all the different levels.”

Tuberculosis does not restrict itself to one host population, and all the members of the MTBC can affect multiple hosts and thus can threaten human and animal health through interspecies transmission. It is of importance to both the animal health and human health sectors as it requires global TB control in all host populations (145). Recognizing the value of cross-sectoral coordination in addressing complex health threats, the FAO, OIE, and WHO formed a Tripartite collaboration in 2010 to develop the concept of One Health and its vision of having a collaborative multidisciplinary work on the health of humans, animals and ecosystems reducing the risk of diseases at the interfaces between them. This FAO–OIE–WHO Tripartite is assessed and updated annually. Their shared “One Health” vision is of “a world capable of preventing, detecting, containing, eliminating and responding to animal and public health risks attributable to zoonoses and animal diseases with an impact on food security through multisectoral cooperation and strong partnerships sharing responsibilities and coordinating global activities to address health risks at the animal-human–ecosystems interfaces” (146). The WHO, in 2014 adopted their “End TB” goals of ending the TB epidemic by 2030, achieving a 95% reduction in TB deaths and a 90% reduction in TB cases by 2035, and have determined that a comprehensive approach is needed which includes new and more effective vaccines, as well as improved diagnostics and treatment (147).

In October 2017, the first-ever roadmap to combat zoonotic TB, i.e., TB in animals and its transmission to humans, was launched at the 48th Union World Conference on Lung Health (148). This multidisciplinary roadmap developed by four groups comprising the WHO, the OIE, the FAO, and the International Union Against Tuberculosis and Lung Disease (The Union) represents a milestone in the fight against TB in both people and animals. It builds on the United Nations Sustainable Development Goals to improve health worldwide including a target to end the global TB epidemic by 2030 as defined by the WHO in the End TB Strategy (147) which acknowledges that people at risk of zoonotic TB are a neglected population deserving greater attention. The roadmap uses a “One Health approach” to address the health risks of TB across sectors to reduce the burden of zoonotic TB, to save lives and secure livelihoods. The roadmap sets out 10 priorities to tackle zoonotic TB under 3 main headings (1) to improve the scientific evidence base, (2) to reduce transmission at the animal–human interface, and (3) to strengthen intersectoral and collaborative approaches. In agreement with others (16, 19, 21), the roadmap acknowledges that the human burden of disease cannot be reduced without management of the animal reservoir, but it also stresses that major technical and scientific obstacles, including the development of novel and affordable diagnostic tests, must be overcome and validated as effective under field conditions.

Indeed, the transmission of *M. tuberculosis* from human-to-animal-to-human still occurs, and is an ongoing risk, especially in countries where there is close interaction of humans with animals and, is of particular public health concern, in places such as zoos, circuses, and exotic animal facilities where there may be contact between TB-susceptible animals and humans (30–37). Where there is no effective eradication programme operational in cattle, the routine presence of *M. tuberculosis* in samples from multiple cattle raises the possibility of human-to-cattle-to-human transmission and possible adaptation of strains of *M. tuberculosis* in bovine or other animal tissues underlining the importance of adopting effective TB control and eradication programmes in humans and livestock alike (38–41).

Discussions on “Does risk to humans justify high cost of fighting bTB?” demands that the “Benefits of stemming bTB need to be demonstrated” and claims that bTB “control in cattle is irrelevant as a public health policy” (149–151) serve to demonstrate that the hard-learnt lessons of history have largely been forgotten and that the “One Health” message on the risk posed to human health (144) is not penetrating to all parties. Pasteurization of bovine milk alone will likely not be sufficient to protect public health if multispecies-based TB controls cease and/or if a strain of *M. tuberculosis* adapted by passage through bovines or some other domesticated or wild species develops that is even more virulent for man and/or has also developed antimicrobial resistance. Strategic exchange of data and discussions involving both veterinary and public health authorities would strengthen TB surveillance in both animal and human populations (152).

Wildlife conservation and ecosystem preservation can also benefit from a One Health approach. The Wildlife Conservation Society recognizes the inextricable linkage between conservation, human health, and the health of wild and domestic animals (146). A single pathogen could wipe out the last populations of an endangered species and, in turn, threaten the stability of local human populations. TB is among its “deadly dozen” potentially lethal diseases that could spread. Economic, environmental and ecological conditions can promote contact between wildlife and livestock and in turn increase transmission of TB at the livestock–wildlife interface. Numerical increases or spatial concentrations of the wildlife population can increase the competition between wildlife and livestock for water and food thus potentially promote the spread of TB directly or indirectly due to the ability of mycobacteria to survive outside a host for a period. Studies have demonstrated that animals in wildlife reservoirs are capable of excreting mycobacteria which can serve as a source of infection to other animals (153, 154). The Wildlife Conservation Society’s concept “One World, One Health™” program is a holistic initiative that manages human, wildlife and domestic animal health issues according to a fundamental truth—the “One Health” that affects all is the health of the planet’s ecosystems and advises that “the monitoring of wildlife health provides us with a sensitive and quantitative means of detecting changes in the environment. Without wildlife, we may not see what is coming until a crisis has occurred. Wildlife monitoring provides a new lens to see what is changing around us to help governments, world health agencies, and regional communities detect threats and mitigate them before they become health crises” (34, 35, 154).

## Conclusion

In conclusion, as Sternberg-Lewerin (145) succinctly put it, “A One Health approach is clearly warranted for TB. The disease has similarly serious consequences for humans and a broad range of animal species, and it has been strongly advocated as a One Health issue.” TB and specifically zoonotic TB was, and still is, important; ending the TB epidemic in humans and the eradication of TB in cattle and other animals are worthwhile goals for human health, zoonosis, animal welfare and socioeconomic reasons and ideally suited for a One Health approach requiring human medical and veterinary interdisciplinary/multidisciplinary collaborative action. Sharing skills and resources, increasing interaction between public health and veterinarians particularly in resource-limited situations, can raise awareness of the “shared risk” of TB between humans and animals and would help to reduce unnecessary duplication of effort (143, 155). To successfully control TB, all causes of TB, all members of the MTBC must be tackled in all species in which TB occurs. To ignore a reservoir affected species and the lessons of history is to court disaster.

The pioneer scientists who revolutionized the diagnosis of this disease over a hundred years ago were remarkable, indeed so remarkable that it remains a challenge for today’s scientists to develop a “better test” or a “better” test reagent. The WHO has determined that “major technical and scientific obstacles will need to be overcome, with validation of effectiveness under field conditions. This will require the development of affordable diagnostic tests in parallel to differentiate infected from vaccinated animals.” The target set in the roadmap (148) for the availability of new diagnostics assays for livestock is 2025. However, until this is achieved the tuberculin skin test will necessarily remain the most widely used means of determining the TB infection status of live domestic animals. The necessity to reduce the burden of zoonotic TB, to save lives and secure livelihoods is far too important to await the possible development of novel diagnostic assays for livestock before renewing efforts to eliminate infection in livestock. Tuberculin tests are safe to use and the choice of which type of tuberculin test is determined by the ecosystem in which it will be used. The challenge therefore is to ensure that the tuberculin on the market continues to meet the standards required. Tuberculin potency is critical to test performance and the accurate determination of potency is therefore particularly important. The sale and use of substandard potency tuberculins should no longer be permitted. The skin test needs to remains available, with good Se and Sp, to those far from sophisticated laboratories and with few resources so that it may continue to play a role in TB control in livestock alongside pasteurization of milk for human consumption and public health measures to protect human health and livelihoods. In addition, as suggested by the roadmap for zoonotic TB “the role of wildlife reservoirs, and potential approaches for control through targeted vaccination, could also be further investigated to find sustainable solutions for combatting the disease while safeguarding wildlife conservation.”

## Author Contributions

MG and AD conceived the study. MG, DC, and DB carried out the literature search and compiled the data. MG drafted the preliminary manuscript. All the authors participated in reviewing, editing, read and approved the final draft, and collaborated in producing the final version.

## Conflict of Interest Statement

The authors declare that this review was conducted in the absence of any commercial or financial relationships that could be construed as a potential conflict of interest.
